# A novel structure associated with aging is augmented in the DPP6-KO mouse brain

**DOI:** 10.1186/s40478-020-01065-7

**Published:** 2020-11-23

**Authors:** Lin Lin, Ronald S. Petralia, Ross Lake, Ya-Xian Wang, Dax A. Hoffman

**Affiliations:** 1 Molecular Neurophysiology and Biophysics Section, Program in Developmental Neuroscience, Eunice Kennedy Shriver National Institute of Child Health and Human Development, 35 Lincoln Drive, MSC 3715, Building 35, Room 3C-905, Bethesda, MD 20892-3715 USA; 2 Advanced Imaging Core, National Institute on Deafness and Other Communication Disorders, Bethesda, MD 20892-3715 USA; 3 Laboratory of Genitourinary Cancer Pathogenesis, National Cancer Institute, National Institutes of Health, Bethesda, MD 20892 USA

**Keywords:** DPP6, Alzheimer’s disease, Aging dementia, Presynaptic terminals

## Abstract

In addition to its role as an auxiliary subunit of A-type voltage-gated K^+^ channels, we have previously reported that the single transmembrane protein Dipeptidyl Peptidase Like 6 (DPP6) impacts neuronal and synaptic development. DPP6-KO mice are impaired in hippocampal-dependent learning and memory and exhibit smaller brain size. Using immunofluorescence and electron microscopy, we report here a novel structure in hippocampal area CA1 that was significantly more prevalent in aging DPP6-KO mice compared to WT mice of the same age and that these structures were observed earlier in development in DPP6-KO mice. These novel structures appeared as clusters of large puncta that colocalized NeuN, synaptophysin, and chromogranin A. They also partially labeled for MAP2, and with synapsin-1 and VGluT1 labeling on their periphery. Electron microscopy revealed that these structures are abnormal, enlarged presynaptic swellings filled with mainly fibrous material with occasional peripheral, presynaptic active zones forming synapses. Immunofluorescence imaging then showed that a number of markers for aging and especially Alzheimer’s disease were found as higher levels in these novel structures in aging DPP6-KO mice compared to WT. Together these results indicate that aging DPP6-KO mice have increased numbers of novel, abnormal presynaptic structures associated with several markers of Alzheimer’s disease.

## Introduction

DPP6 is a type II single pass transmembrane protein expressed in brain [35, 64], and well known as an auxiliary subunit of the Kv4 family of voltage-gated K^+^ channels [45], which play a crucial role in neuronal excitability and plasticity [23, 65]. We previously reported a surprising role for DPP6 in hippocampal synaptic development and function that is apparently independent of Kv4.2 [36, 37]. Clinically, the DPP6 gene has been associated with numerous intellectual and neurodevelopmental disorders [6, 17, 33, 41, 43, 47, 53, 55, 56]. An important recent study showed DPP6 to be a novel gene in dementia, finding enhanced rare variants and nonsense, frameshift and missense mutations in early Alzheimer’s disease (AD) and frontotemporal dementia patient cohorts [9]. Another finding by Zelaya et al. [77] demonstrated an olfactory progressive proteome modulation in AD, which shows olfactory dysfunction in up to 90% of patients. They report a specific late reduction of DPP6 in the olfactory bulb of patients with AD. Overall, these findings provided basic information for understanding possible roles of DPP6 in aging and the pathophysiology of diseases such as AD/dementia.

AD is the most common form of major neurocognitive disorder (dementia). It is a progressive neurodegenerative disease associated with memory impairment and cognitive decline. Currently, definitive diagnoses of AD require post-mortem analysis and there are no curative therapies for AD [7, 10, 57]. Diagnostic hallmarks include the abnormal accumulation of extracellular amyloid-β peptide (Aβ, amyloid plaques), intracellular neurofibrillary tangles composed of phosphorylated tau (pTau), and neuronal loss. These abnormalities that characterize AD are particularly prevalent in the hippocampus such that “hippocampal atrophy is one of the earliest detectable symptoms of ongoing neurodegeneration” [42]. Aβ and pTau pathologies are associated with neuroinflammation characterized by activation of microglia and astrocytes and elevated levels of proinflammatory cytokines [50, 60].

In both human patients with early AD and in mouse models of AD, Aβ accumulation precedes and promotes tau pathology. In human brains with early AD, intraneuronal Aβ42 accumulation in hippocampal cell bodies precedes hyperphosphorylation of tau [20, 21], and in a common mouse model of AD (3xTg-AD mouse), intraneuronal Aβ accumulation in cell bodies also precedes tau hyperphosphorylation [48, 49]. Microglia activation and proliferation around amyloid plaques is also a key feature in AD pathogenesis.

Tau proteins stabilize microtubules and play a role in axonal transport and neurite outgrowth [4, 5, 11, 63, 66]. These functions of tau can be modulated by phosphorylation that can be both physiological and pathological to the cell. There is significant evidence that a disruption of normal phosphorylation events results in tau dysfunction in neurodegenerative diseases such as AD, and is a contributing factor to the pathogenic processes [3, 25]. The resulting abnormal tau accumulation in AD/dementia may lead, for example, to various axonopathies such as axonal swellings [38, 70].

In this study, we examined the cell architecture and proteins associated with aging in the hippocampus of DPP6-KO mice, and discovered unique clusters of large NeuN-labelled puncta concentrated throughout the hippocampal CA1 region that were larger and more abundant and expressed at earlier ages in DPP6-KO mice compared to WT. These structures partly colocalized with a number of proteins associated with AD, including Aβ, pTau, α-synuclein and chromogranin A. Labeling for a number of markers of AD was higher in the puncta of the DPP6-KO compared to WT, including Aβ, pTau, α-synuclein, and APP. Immunocytochemical and ultrastructural methods showed that these structures are derived from abnormal swollen presynaptic terminals and are associated with postsynaptic dendrites and synapses. We also found immunocytochemical and biochemical evidence for substantial gliosis. Our findings demonstrate that DPP6, in addition to its effects on early synapse development appears to be important for the maintenance of synaptic function during aging as its loss leads to an increase in abnormal synaptic structures associated with physical and biochemical markers for dementia.

## Materials and methods

### Animals

DPP6-KO and WT mice were weaned at 3 weeks of age, genotyped via PCR, and housed 3–4 mice per cage. Mice were housed under a 12-h light/dark cycle with the lights off at 18:00. We only used male mice in all experiments. DPP6-KO mice were originally from Dr. B. Rudy at NYU School of Medicine, New York, and WT C57BL/6 were originally from Jackson Laboratory. All animal procedures were performed in accordance with guidelines approved by the National Institute of Child Health and Human Development Animal Care and Use Committee and in accordance with NIH guidelines.

### Electron microscopy

Hippocampi were prepared for transmission electron microscope (TEM) study as described previously [32]. Briefly, WT and DPP6-KO, 12-month old mice were fixed in 4% paraformaldehyde plus 2% glutaraldehyde (Electron Microscopy Sciences (Hatfield, PA; EMS) in phosphate buffer (PB), then washed in PB and vibratomed at 150 µm. Slices were washed in cacodylate buffer and placed in 1% osmium tetroxide in cacodylate buffer, washed again in cacodylate buffer, and dehydrated in an ethanol series with 1% uranyl acetate added to the 50% ethanol. Tissue was placed in propylene oxide (PO), and then in an epon/PO mix and finally in pure epon; then samples were embedded and hardened in an oven at 64 °C. Thin sections (60 nm) were placed on single slot, formvar/carbon coated nickel grids (EMS), stained with uranyl acetate and lead citrate, and examined in a JEOL JEM2100 TEM (Peabody, MA). Images were taken from 2 WT and 2 KO mice, in all areas of the CA1, as well as in the molecular layer of the dentate gyrus.

Mouse hippocampi used for postembedding immunogold localization were prepared as described previously [22, 36, 52, 65]. Briefly, WT and DPP6-KO, 12-month old mice were perfused with phosphate buffer, followed by perfusion with 4% paraformaldehyde + 0.5% glutaraldehyde in phosphate buffer. Fixed brains were vibratomed at 350 μm, then cryoprotected in glycerol overnight, frozen in a Leica EM CPC (Vienna, Austria), and processed and embedded in Lowicryl HM-20 resin in a Leica AFS freeze-substitution instrument. Thin sections were incubated in 0.1% sodium borohydride + 50 mM glycine in Tris-buffered saline plus 0.1% Triton X-100 (TBST). Sections were immersed in 10% normal goat serum (NGS) in TBST, and primary antibody in 1% NGS/TBST (overnight), then incubated with immunogold-conjugated secondary antibodies (Ted Pella, Redding, CA, USA) in 1% NGS in TBST with 0.5% polyethylene glycol (20,000 MW), and stained with uranyl acetate and lead citrate. For double-immunogold labeling, the 2 primary antibodies were incubated together, and also the 2 secondary immunogold antibodies were incubated together.

**Fig. 1:**
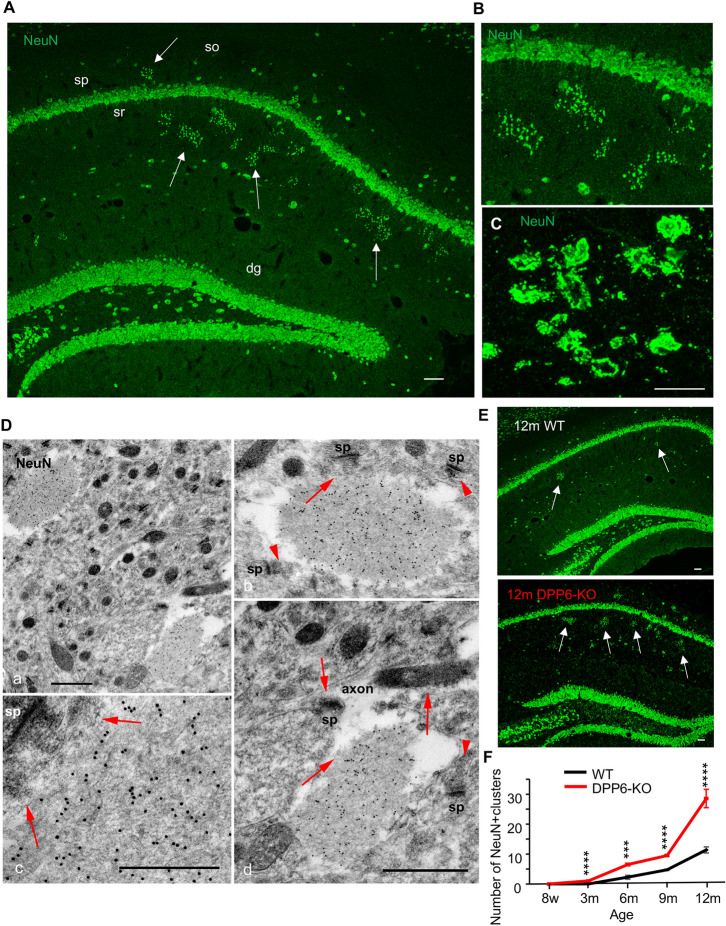
Novel large clusters of NeuN^+^ puncta/swellings are found in the hippocampus of DPP6-KO mice. **A** In the CA1 region of the hippocampus of 12-month old DPP6-KO brain sections, immunofluorescence labeling for NeuN (green, 1:500, Millipore) is concentrated not only in the neuronal somas but also in novel structures made of enlarged, variable puncta formed in clusters (arrows). Scale bar = 100 μm, dg, dentate gyrus; so, stratum oriens; sp, stratum pyramidale; sr, stratum radiatum. **B** A higher magnification image of one of the clusters shown in **A**. **C** Even higher magnification of a single cluster from another section. Scale bar = 5 μm. **D** Immunogold labeling of NeuN (15 nm) in the CA1 stratum radiatum of the hippocampus of a WT 12-month old mouse. **Da** is a low magnification including two NeuN^+^ swellings. The top swelling is shown in higher magnification in **Db, c** and the bottom swelling is shown in **Dd**. Note how the immunogold labeling is concentrated mainly in the two adjacent swellings, which show a fibrous interior and traces of presynaptic vesicles (active zone; arrows) on the periphery, associated with postsynaptic spines (sp). The structure of these vesicles resembles those seen in adjacent separate presynaptic terminals (arrowheads); in **Db**, the cluster of presynaptic vesicles in the lower left (arrowhead) probably is a presynaptic zone of the swelling also, but the connection is not distinct in the image. In **Dd**, the NeuN^+^swelling extends off of an axon with presynaptic vesicles that makes a synapse with a spine in the image. (**Da, d**; the scale bar in **d** also applies to **b**) or 500 nm in **Dc**. Compare to examples from a DPP6-KO mouse shown in the Additional file 1: Fig. S1. And compare the presynaptic active zones in the periphery of the swellings in Figs. 1D, 3, and Additional file 1: Fig. S1. **E** These novel NeuN-labeled clusters are observed more in 12-month old DPP6-KO mice than in WT. Scale bar = 100 μm. **F**: Graph comparing the number of novel clusters in the CA1 region of the hippocampus during development from 8-week to 12-month old mice in WT and DPP6-KO mice. From 8-week to 12-month old, labeling in aged DPP6-KO mice is significantly increased compared to WT (n = 8–38 images per age from 3 to 8 mice each WT and DPP6-KO.*** = *p* < 0.001, **** = *p* < 0.0001)

Two mice each from WT and DPP6-KO were studied for NeuN (1:50, rabbit, MilliporeSigma) and NeuN (1:50–1:100, mouse, Millipore) antibodies, and for antibodies to MAP2 (1:100, mouse, Millipore), synaptophysin (1:7–1:10, mouse, Sigma), APP (1:25, rabbit, Abcam), and Aβ-amyloid (1:25–1:50, rabbit, MilliporeSigma). NeuN immunogold labeling was studied in either single labeling or double labeling with synaptophysin or MAP2; the latter 2 antibodies were studied only in the double labeling. The mouse NeuN immunogold labeling (1/100 and 15 nm gold) shown in Fig. 1 was taken from a double labeling study with rabbit GFAP (1/200 and 5 nm gold), which labeled mainly the glial cell somas and associated large processes (data not shown). Additional immunogold studies with antibodies to AT8 (1:25, mouse, Thermo Fisher), Aβ amyloid (1:50, mouse, BioLegend) and another synaptophysin (1:7, SP15, mouse, MilliporeSigma) produced only rare (background) gold labeling and thus were controls for the immunogold technique. Most successful immunogold labeling studies concentrated on the NeuN^+^ swellings, which were difficult to find due to their scattered distribution and required extensive searching on the EM sections.

### Immunofluorescence and immunohistochemistry

Anesthetized mice were perfused with PBS buffer and then with 4% paraformaldehyde (PFA) in PBS. The collected brains were placed in 4% PFA for 24 h and then equilibrated in 30% sucrose for 24 h. A series of equidistant floating 30 μm or frozen 7 μm coronal sections were prepared. First, sections were incubated in blocking buffer (10% Normal goat serum and 0.3% Triton X-100 in PBS) for 30 min at RT. Thereafter, samples were incubated overnight with the primary antibody at 4 °C and then were incubated with the appropriate fluorescent probe-conjugated secondary antibodies for 1 h at RT. Nuclei were counterstained with DAPI. Immunofluorescence-labelled slides were imaged using either a Zeiss 710 or 880 confocal microscope; or the entire brain section or hippocampus was scanned using a Carl Zeiss AxioScan.Z1 slide scanner with a 20 × objective (Plan-Apochromat, NA 0.8) and a Colibri7 LED illumination source. We used ImageJ for IF quantification with blinded images.

### Antibodies

**Table:** 

Name	Species	Company	Catalog#	IHC
Amyloid Precursor Protein	Rabbit	Abcam	ab32136	1:500
beta-Amyloid 1–16	Mouse	BioLegend	803001	1:1000
beta-Amyloid 1–42	Rabbit	MilliporeSigma	Ab5078P	1:400
alpha-Synuclein	Rabbit	Novus	NBP2-15365	1:400
Chromogranin A	Rabbit	Novus	NB120-15160SS	1:500
GAD67	Mouse	Abcam	ab26116	1:2000
GFAP	Mouse	Chemicon	MB360	1:1000
GFAP	Rabbit	DAKO	Z033429-2	1:2000
Iba1 (ICC IHC)	Rabbit	Wako	019-19741	1:800
MAP-2	Mouse	Millipore	MAB-3418	1:500
MAP-2	Rabbit	Millipore	Ab-5622	1:500
NeuN	Mouse	Millipore	MAB377	1:1000
NeuN	Rabbit	MilliporeSigma	ABN78	1:500
Synapsin I	Rabbit	Novus	NB300-104	1:200
Synaptophysin	Mouse	Sigma	S-5768	1:500
PHF-Tau at Thr231 (AT180)	Mouse	Thermo Fisher	MN1040	1:200
Ubiquitin	mouse	Abcam	AB7254	1:1000
VGLUT1	Guinea pig	Millipore	AB5905	1:500

### Imaging and statistical analyses

We used ImageJ to measure the intensity of each NeuN+ large punctum. First, we outlined the punctum based on the NeuN + labeling; then, we switched to the other marker channel to use the same punctum shape to measure the mean intensity and surface area. The n values and details of controls and comparisons used for statistical analyses are described for each experiment in the corresponding figure legends section. Statistical analyses were performed using GraphPad Prism 8. We used one-way or two-way ANOVA and Student’s *t* test. All results are presented as the mean ± SEM.

## Results

### A novel structure in aging mice develops earlier and is more abundant in the DPP6-KO

During the performance of Immunofluorescence (IF) staining with NeuN antibody to characterize the neurons in the hippocampus, of 12-month DPP6-KO and corresponding WT mice, we noted clusters of large puncta with NeuN labeling in the CA1 region of DPP6-KO mice (Fig. 1A–C). NeuN is a neuron-specific RNA-binding protein, Rbfox3, with at least 2 subtypes including one (46-kDa) that is mainly nuclear and another (48-kDa) that is mainly cytoplasmic [14]. Most studies report cytoplasmic localization in neuron somas and some dendrites, but Luca et al. [40] also found axonal localization in the occipital cortex of humans with HIV-associated cognitive impairment. Subsequent electron microscope studies using immunogold labeling (15 nm) for NeuN in the CA1 stratum radiatum of the hippocampus revealed that the gold labeling was highly concentrated mainly in large swellings, with relatively few gold particles found in the surrounding neuropil (Fig. 1D). The images show immunogold labeling that was concentrated mainly in two adjacent swellings, which had fibrous interiors and traces of presynaptic vesicles (active zone; arrows) on the periphery, associated with postsynaptic spines (sp). Note especially Fig. 1Dd, in which the synaptic region of the swelling was seen extending from an axon. We further wondered when these novel structures form. We examined mice at 5 different age points in a range of ages from 8-weeks to 12 months for WT and DPP6-KO mouse hippocampi (Fig. 1E, F). In 8-week adult mice, we didn’t find any novel NeuN^+^ structures in either WT or DPP6-KO mice. A few of these were observed in 3-month old DPP6-KO mice but not in WT. Clusters increased up to 12 months of age in both DPP6-KO and WT mice although there were nearly threefold more found in DPP6-KO compare to WT (Fig. 1F, *p *< 0.0001). Figure 1E shows an example of the labeling in 12-month old WT and DPP6-KO mice.

**Fig. 2:**
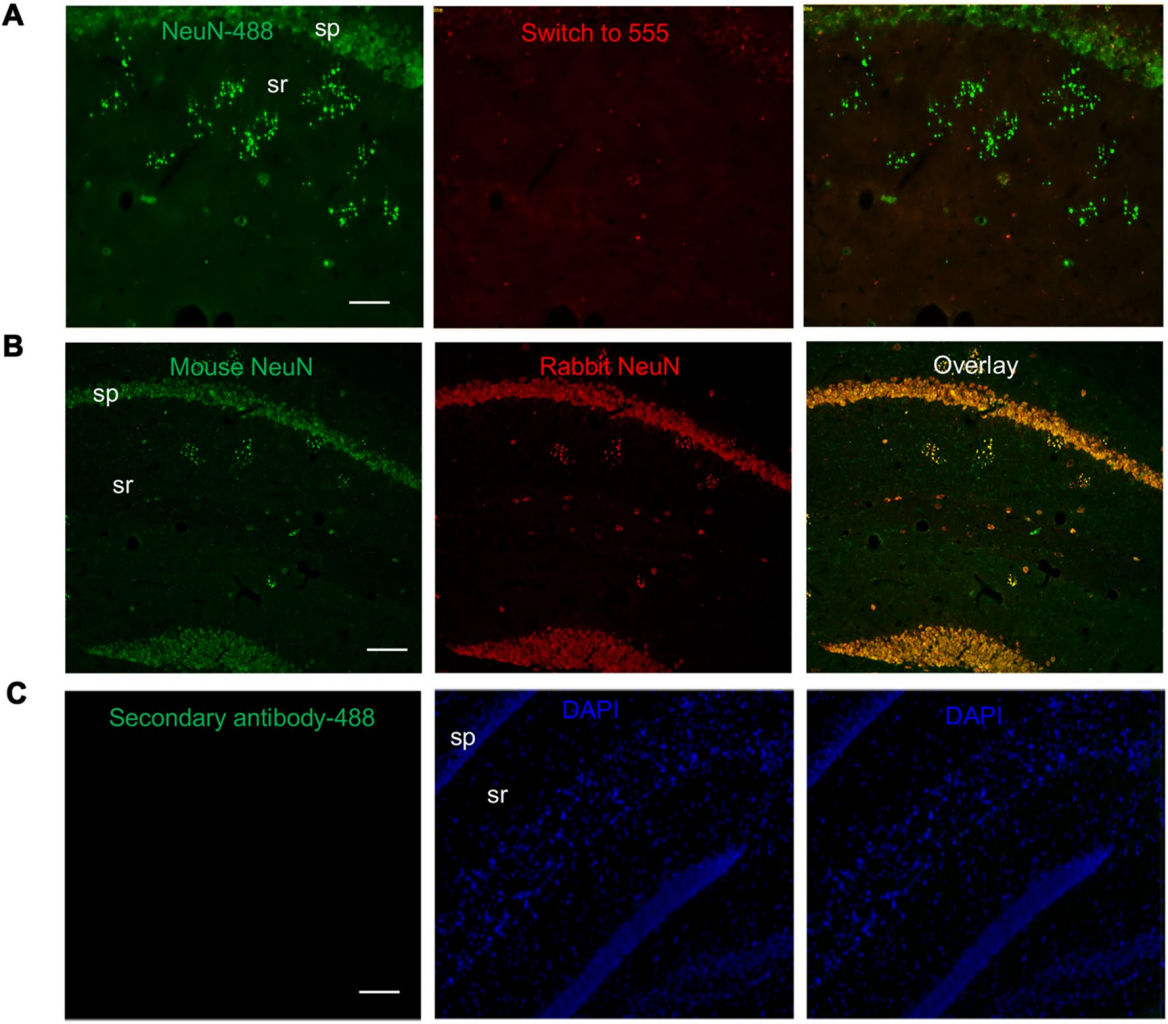
Controls for immunofluorescence labeling. **A** IF labeling of NeuN^+^ puncta with Alexa fluor-488 goat anti-Rabbit IgG secondary antibody (1:800, ThermoFisher). The NeuN labeled puncta only can be observed at 488, and not at 555, showing that the NeuN labeling is not caused by autofluorescence. **B** Double labeling showing the colocalization of NeuN mouse antibody (green) and rabbit antibody (red). Scale bar = 50 μm. **C** IF labeling only with Alexa fluor-488 goat anti-Rabbit IgG secondary antibody (1:800, ThermoFisher) control; without the primary NeuN antibody, the puncta cannot be observed. Scale bar = 50 μm. sp, stratum pyramidale; sr, stratum radiatum

We confirmed that this NeuN labeling is not caused by autofluorescence (Fig. 2a) and it is consistent between two different mouse and rabbit species of NeuN antibodies (Fig. 2b). We also confirmed that this labeling is absent in sections stained with the control secondary antibody only (Fig. 2c).

### The novel structure shows characteristics of abnormal presynaptic terminals

**Fig. 3:**
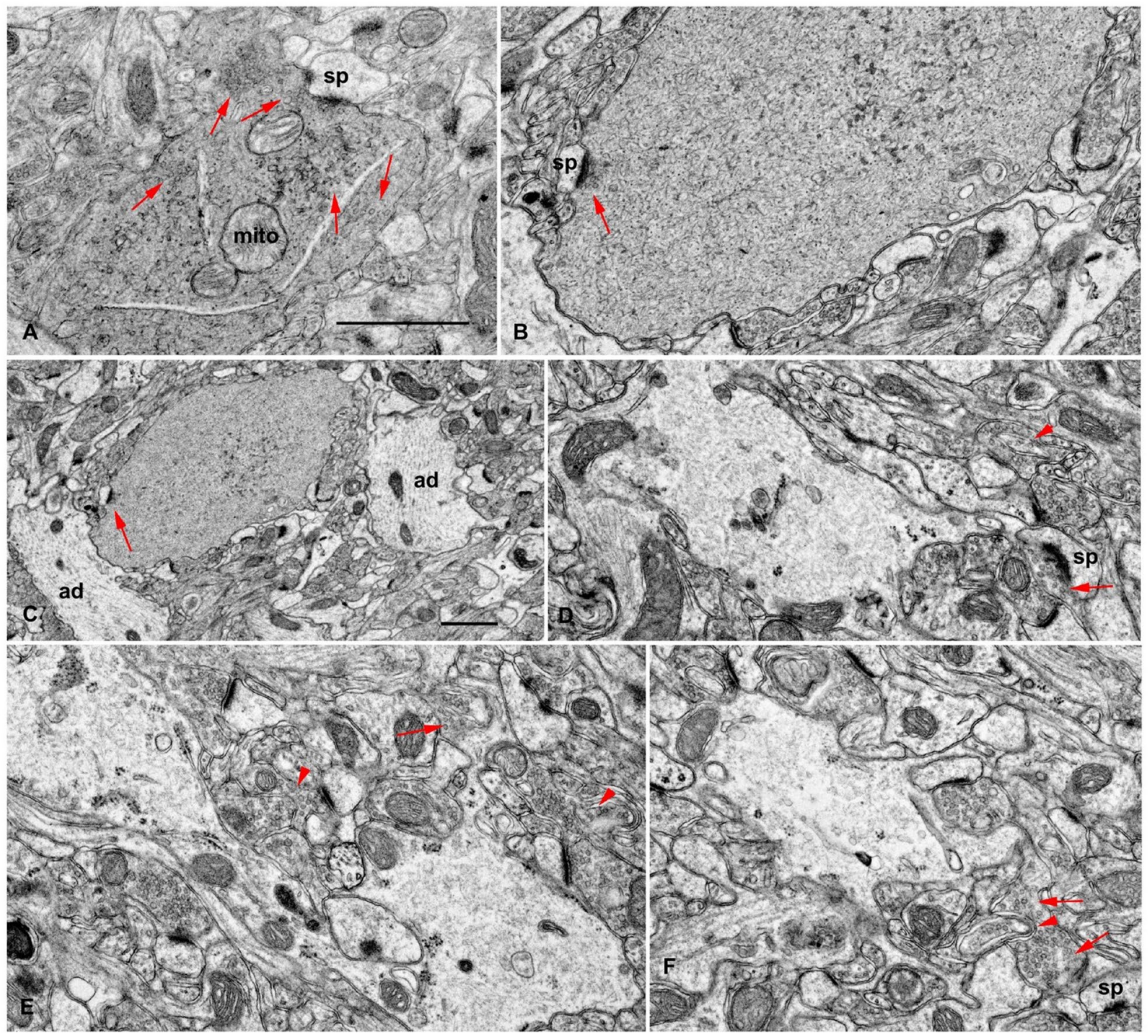
Ultrastructural study of large swellings seen in the CA1 region of the hippocampus of 12-month-old mice These likely correspond to the large puncta found in the novel clusters labeled with immunofluorescence for NeuN (Fig. 1a–c). All appear to be derived from presynaptic terminals, and they may represent approximately 3 stages in the formation of the swellings. **a** Stage 1: The swelling in **A** shows central mitochondria (mito) and large clusters of presynaptic vesicles (arrows) including one cluster at an active zone on a spine synapse (sp). The cytoplasm is dense and full of thick fibrils. **B**, **c** Stage 2: The swelling in **B** and ***c*** is filled with thick fibrils but has very few presynaptic vesicles; a group (arrow) can be seen at an active zone of a spine synapse (sp). **A**–**C** are from the CA1 stratum radiatum (**A**, KO; **B, C**, WT). **D**–**F** -Stage 3: Many of the swellings are light with no evident presynaptic vesicles and with thin fibrils. In these 3 examples from the CA1 stratum oriens of a DPP6-KO mouse, the periphery of the swelling is very convoluted, and it extends thin processes around the surrounding synapses (similar in appearance to glial processes). However, in all 3 images, one of the processes from the swelling appears to be continuous with a terminal filled with presynaptic vesicles (arrows). **D–F** also shows distinctive invaginating presynaptic terminals (arrowheads). Scale bars in **A** and **C** are 1 μm, and the one in **A** is for all other micrographs. ad, apical dendrite of pyramidal neuron

We then performed ultrastructural studies using transmission electron microscopy (TEM), and we documented novel, large swellings in the CA1 region of the 12-month DPP6-KO mice (Fig. 3). Thus, the large swellings seen with both ultrastructural and immunogold EM studies may represent the large NeuN^+^ puncta seen with IF. The presence of high concentrations of NeuN, seen with both IF and EM, confirm that these structures are derived from neurons. Further, the structural evidence suggests that these swellings were formed from presynaptic terminals, represented in approximately 3 stages in the formation of the swellings. Stage 1—seen in Fig. 3a: In this example, the swelling shows central mitochondria (mito) and large clusters of presynaptic vesicles (arrows) including one cluster at an active zone on a spine synapse (sp). The cytoplasm is dense and full of thick fibrils. Stage 2—seen in Fig. 3b, c: Here the swelling is filled with thick fibrils but has very few presynaptic vesicles; a group (arrow) can be seen at an active zone of a spine synapse (sp). Presynaptic active zones also are seen in the periphery of some swellings labeled with immunogold for NeuN (Fig. 1D and Additional file 1: Fig. S1). In Fig. 1D, clusters of presynaptic vesicles (arrows in 1D*b*-*d*) are found in the peripheral area of the swelling and form at an active zone with a postsynaptic spine (sp). Similar examples of presynaptic vesicles (p) at active zones in the swelling periphery and opposite postsynaptic spines (sp) are seen in Additional file 1: Fig. S1A,B. Also, by stage 2, organelles tend to be lost from the swelling; the remainders of dysmorphic mitochondria can be seen in the swelling in Additional file 1: Fig. S1C. Stage 3—Fig. 3d, f: Many other swellings are light with no evident presynaptic vesicles and with thin fibrils. In these 3 examples from the CA1 stratum oriens of a DPP6-KO mouse, the periphery of the swelling is very convoluted, and it extends thin processes around the surrounding synapses (similar in appearance to glial processes). However, in all 3 images, one of the processes from the swelling appears to be continuous with a terminal filled with presynaptic vesicles (arrows in Fig. 3d, f). Overall, these results suggest that the novel large clusters of NeuN^+^ puncta seen with IF are the large NeuN^+^ swellings seen with EM.

**Fig. 4:**
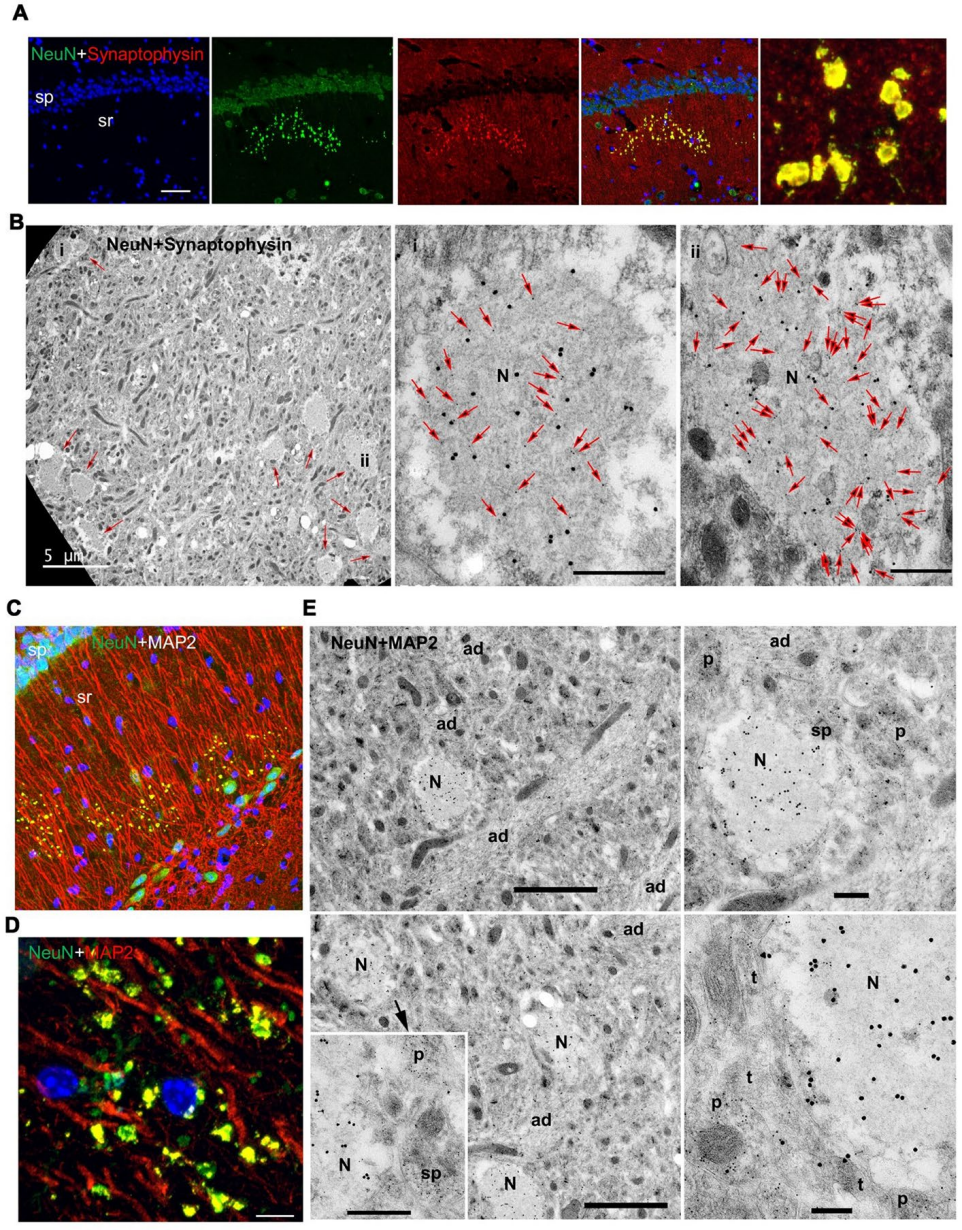
The novel clusters of NeuN^+^ puncta are formed from synaptophysin-positive presynaptic, swollen terminals and MAP2 positive postsynaptic structures. **A** In the CA1 region of the hippocampus of a 12-month old DPP6-KO, immunofluorescence for NeuN (green, 1:500, Millipore) and synaptophysin (red, 1:500, Sigma) show complete colocalization in clusters of the novel large puncta. On the right is a high magnification image showing the colocalized NeuN and synaptophysin in the individual, large puncta of the cluster. Scale bar = 50 μm. sp, stratum pyramidale; sr, stratum radiatum. **B** Immunogold labeling for NeuN and synaptophysin in the deep region of the CA1 (probably stratum lacunosum-moleculare) of the DPP6-KO mouse. 20 nm gold for NeuN is concentrated within the large swellings (arrows in low magnification image; N in high magnification images, **Bi** and **Bii**), where it colocalizes very well with 10 nm gold (arrows in high magnification images; *all* 10 nm gold particles in the image are indicated with the arrows) for synaptophysin. Both gold sizes are relatively uncommon outside of the swellings. The positions of swellings in **Bi** and **Bii** are indicated on the low magnification, left micrograph, which has at least 10 swellings (arrows). Scale bars in **Bi** and **Bii** are 500 nm. **C, D** In the CA1 of the 12-month old DPP6-KO hippocampus, immunofluorescence for NeuN (green, 1:500, Millipore) and MAP2 (red, 1:500, Millipore) are partly colocalized in the large novel puncta clusters, with MAP2 on the periphery of most NeuN^+^ puncta and throughout some of them, as evident in the higher magnification (**D**); MAP2 also is evident in the apical dendrites of the CA1 pyramidal neurons that overlap with the clusters. Nuclei were counterstained with DAPI in blue. Scale bar = 5 μm. sp, stratum pyramidale; sr, stratum radiatum. **E** Immunogold labeling in the CA1 stratum radiatum of the 12-month DPP6-KO mouse. 20 nm gold for NeuN is concentrated within the large swellings (N), while 10 nm gold for MAP2 is mostly absent from the swellings except for a few small clusters. In contrast, the MAP2 labeling is highest in the large apical dendrites (ad) and smaller postsynaptic structures (p) including spines (sp); but presynaptic terminals (t) show little labeling. The right micrographs show higher magnifications of the swelling in the upper left micrograph. The lower left inset is magnified from the lower left micrograph. Scale bars on the left are 2 micrometers, the bar in the inset is 500 nm, and the right scale bars are 400 and 200 nm for the top and bottom micrographs, respectively

We found complete colocalization within the clusters when we performed IF staining with NeuN and the presynaptic marker synaptophysin (Fig. 4a), confirming that the large NeuN^+^ clusters are formed from presynaptic terminals. Similarly, EM immunogold labeling showed that 20 nm gold for NeuN is concentrated in the large swellings and colocalizes very well with 10 nm gold for synaptophysin (Fig. 4b; the arrows in the 2 high magnification images label *all* 10 nm gold particles to illustrate the reciprocal distribution of NeuN and synaptophysin throughout the center of the swelling). Both results suggest that the novel puncta are modified/derived from presynaptic terminals.

**Fig. 5:**
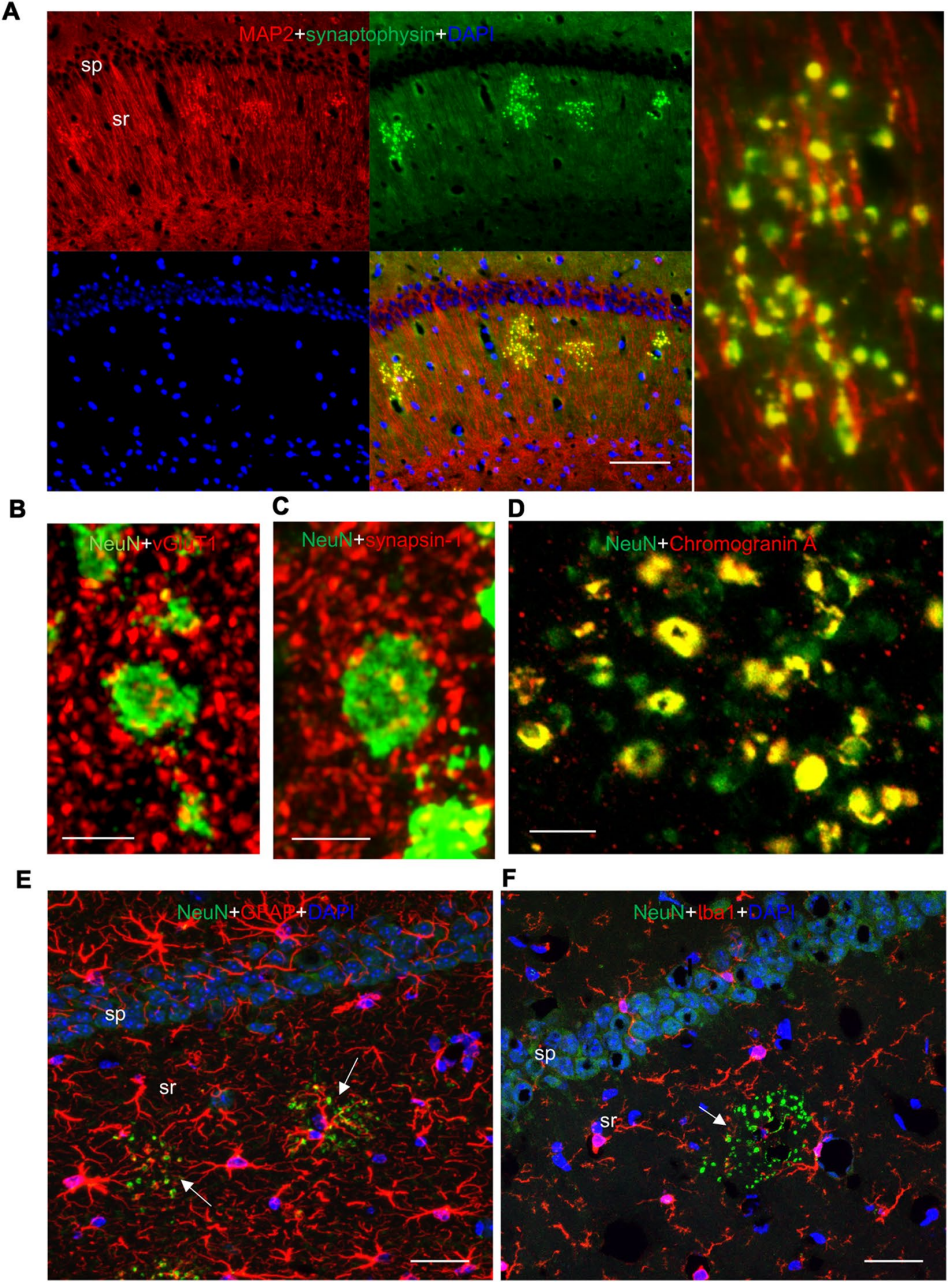
NeuN^+^ clusters have partial colocalization of MAP2 and synaptophysin, strong colocalization with Chromogranin A, presynaptic markers in their periphery, and nearby glia. **A** In the CA1 region of the hippocampus of 12-month old DPP6-KO mice, immunofluorescence shows MAP2 (red, 1:500, Millipore) and synaptophysin (green, 1:500, Sigma) partly colocalized in clusters of puncta; on the right is the high magnification image. Scale bar = 50 μm. **B**, **c** Immunofluorescence staining shows the novel puncta labeled with NeuN and the presynaptic markers vGluT1 (**B**; red, 1:500, Millipore) and synapsin1 (**C** red, 1:200, Novus), **D** The NeuN^+^ novel puncta colocalize very well with Chromogranin A (red, 1:500, Novus; Scale bar = 5 μm). **E, F** The novel puncta are surrounded by astrocytes labeled with marker GFAP (**E** red, 1:2000, DAKO) and (**F**) microglia labeled with marker Iba1 (green, 1:800, Wako). Scale bar = 50 μm. sp, stratum pyramidale; sr, stratum radiatum. Nuclei were counterstained with DAPI in blue

**Fig. 6:**
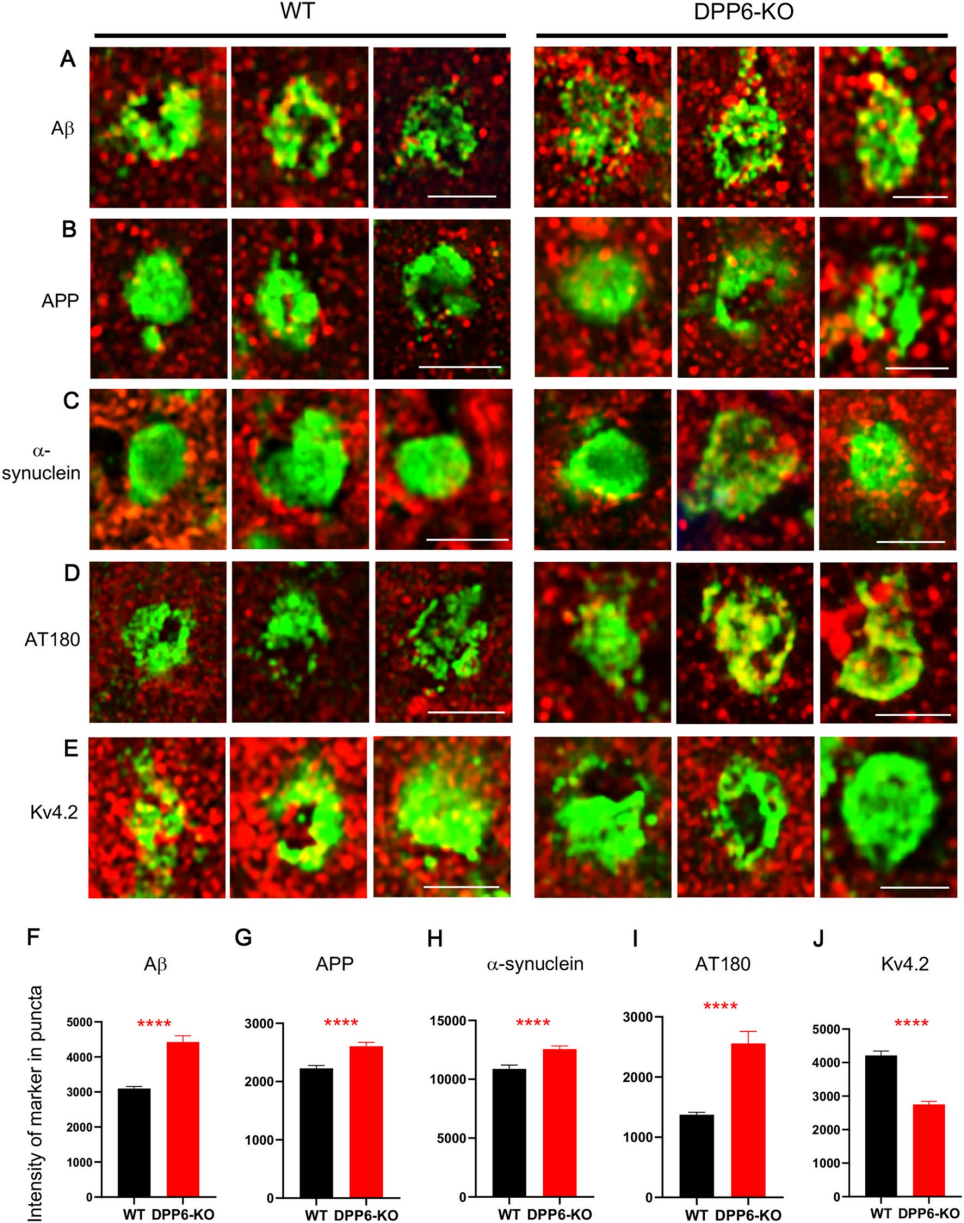
Age and AD-related markers increase and Kv4.2 decreases in NeuN + puncta in the DPP6-KO mice. Labeling of individual NeuN + puncta for Aβ (**A** red, 1:1000, BioLegend), APP (**B** red, 1:500, Abcam), α-synuclein (**C** red, 1:200, Novus), and AT180 (**D** red 1:300, ThermoFisher) is increased in DPP6-KO mice compared to WT (**F** Aβ; WT, n = 267; DPP6-KO, n = 207; **** = *p* < 0.0001; **G** APP; WT, n = 215; DPP6-KO, n = 264; **** = *p* < 0.0001; **H** α-synuclein; WT, n = 129; DPP6-KO, n = 221; **** = *p* < 0.0001; **I** ΑΤ180; WT, n = 192; DPP6-KO, n = 149; **** = *p* < 0.0001;); labeling is mainly in the peripheral regions of the NeuN + puncta. In contrast, labeling for the DPP6-associated protein, Kv4.2, is decreased in the NeuN + puncta of DPP6-KO mice compared to WT (**E,J** WT, n = 116; DPP6-KO, n = 91; **** = *p* < 0.0001). Scale bars = 2 μm

We also performed IF staining for the dendritically enriched marker MAP2. Images showed that MAP2 is only partly colocalized with NeuN clusters of puncta (Fig. 4c, d). Similarly, with 10 nm gold for MAP2, EM immunogold labeling showed partial colocalization with the NeuN^+^ swellings, mainly in postsynaptic structures around the periphery of the swellings (Fig. 4e). IF images also showed that MAP2 and synaptophysin are partly colocalized in the puncta (Fig. 5a). In addition, several presynaptic terminal markers labeled some NeuN + puncta on the periphery of the NeuN^+^ large puncta, including commonly VGluT1 and Synapsin-1 (Fig. 5b, c). But these NeuN^+^ large puncta did not colocalize with antibodies to GAD67, a marker for GABAergic, inhibitory neuronal processes (Additional file 2: 2A). Interestingly, labeling for Kv4.2, a functional binding partner of DPP6, was lower in DPP6-KO NeuN + puncta compared to WT (Fig. 6e, j).

### The novel structure has markers of aging and/or AD

We further characterized by IF the novel clusters with markers that are associated with aging and AD. The NeuN^+^ puncta in the clusters were partially colocalized with Aβ, APP, α-synuclein, and pTau (AT180; Fig. 6a–d) and more fully colocalized with chromogranin A (Fig. 5d). Chromogranin A is a mediator between neuronal, glial and inflammatory mechanisms found in AD, and about 30% of β-amyloid plaques have been shown to co-label with chromogranin A [30]. Interestingly, labeling of the NeuN + puncta for Aβ, APP, α-synuclein, and AT180 was greater in the DPP6-KO compared to WT, and the labeling was concentrated in the peripheral region of the puncta (Fig. 6f–i). However, the puncta were not labeled for ubiquitin, a protein found commonly in amyloid plaques and neurofibrillary tangles in AD (Additional file 2: Fig. S2B). These novel puncta also were surrounded with activated astrocytes (Fig. 5e) and microglia (Fig. 5f).

**Fig. 7:**
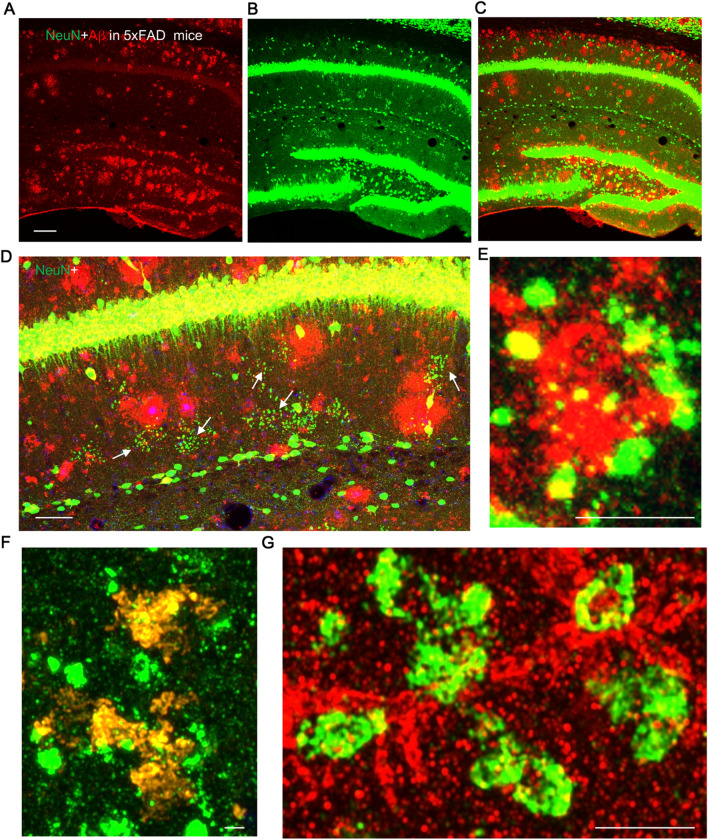
Labeling for Aβ partly colocalizes with NeuN + puncta in 10-month old 5xFAD Alzheimer’s disease mouse hippocampus. The puncta are observed by immunofluorescence labeling for NeuN (green, 1:500, Millipore Sigma; arrows), and the Aβ labeling reveals the plaques (red, 1:1000, Biolegend). Aβ-labeled plaques are most abundant in the hilus and cell body layer of the dentate gyrus and in the stratum oriens, while NeuN^+^ puncta are abundant in the CA1 stratum radiatum and molecular layer of the dentate gyrus (**A**–**C**, and** d**, a medium magnification image). Many Aβ plaques show little or no labeling for NeuN, although NeuN + -clusters of puncta often are contiguous with plaques (**D**). Occasionally, an Aβ plaque is surrounded by NeuN + smaller puncta (**E**), or there may be colocalization of moderate labeling of NeuN within the plaque (**F**), or some substantial accumulations of Aβ-labeling partially colocalize with NeuN + puncta (**G**). Scale bar = 50 μm in panels **A**–**E**; scale bar = 5 μm in panels **F**–**G**

NeuN^+^ puncta were observed in the hippocampus of an AD model mouse. We used 10-month old 5xFAD Alzheimer’s disease mouse brain sections, labeling with IF for NeuN and Aβ antibodies. Aβ-labeled plaque density was highest in the hilus and cell body layer of the dentate gyrus and in the stratum oriens (Fig. 7a–d). The distribution of the NeuN + puncta was actually greater in the 5xFAD mice compared to either the WT or DPP6-KO mice in our study, with abundant puncta in the molecular layer of the dentate gyrus as well as in the stratum radiatum (Fig. 7a–d). Using low magnification confocal images, most Aβ plaques showed little or no direct labeling for NeuN, although plaques and NeuN + -clusters of puncta often were contiguous (Fig. 7d). Using high resolution confocal, we found some examples of Aβ-labeled plaques that were surrounded by NeuN + smaller puncta (Fig. 7e), or in which there was moderate labeling of NeuN within the plaque (Fig. 7f). The latter labeling of the Aβ-labeled plaques for NeuN may be due to the remnants of dystrophic neurites [7, 68]. We also found partial colocalization of labeling for Aβ in or around some NeuN + large puncta (Fig. 7g), as described for the normal 12-month WT and DPP6-KO mice. Thus, there does appear to be some correlation of NeuN + puncta and plaque formation, suggesting that puncta formation may be linked somehow to the overexpression of Aβ in the 5xFAD mice, especially since the NeuN + puncta are more widespread in this AD model compared to the DPP6-KO mice.

## Discussion

### DPP6-KO model reveals a novel, native structure that is plaque-like and may be associated with neurodegeneration

In this study, we have found novel clusters of large puncta in the hippocampal CA1 region in the aging brain. The presence of these clusters was increased in mice lacking the DPP6 protein. NeuN and synaptophysin are highly concentrated and completely colocalized in these structures, as seen with both IF and EM. The presence of synaptophysin in our NeuN^+^ large puncta is consistent with our observations that these structures are modified from presynaptic terminals. This supports the ultrastructural evidence that indicates that the swellings are derived from presynaptic terminals that go through stages of change, gradually losing their evident synaptic vesicles and becoming filled with a fibrous matrix. At least in the intermediate stages of this change, active zones with synaptic vesicles continue to form on the periphery of the swelling, forming synapses with dendritic spines, presumably from the adjacent apical dendrites of the CA1 pyramidal neurons. This is shown in our ultrastructural studies and is consistent with the immunofluorescence labeling of synapsin-1 and VGluT1 found mainly on the periphery of the NeuN + synaptophysin-labeled swellings. Thus, we believe that most of the NeuN and synaptophysin, or at least those portions of these proteins with their antibody epitopes, accumulate in the growing centers of these swellings (along with chromogranin A), forming a matrix material separate from the proteins of the remaining peripheral active zones. Around the swellings, MAP2 is highest in postsynaptic structures including dendrites and their spines [44, 68] that closely cover the swellings, as shown in both IF and EM; both methods also indicate that MAP2 only partially colocalizes with NeuN in portions of the swellings, unlike synaptophysin. The novel puncta are characterized by the accumulation of aggregated Aβ peptides and chromogranin A, and also are colocalized with α-synuclein, and with inflammation-associated glial cells, including both astrocytes and microglia (for reviews of gliosis in AD, see [50, 60]). Like MAP2, Aβ-labeled structures appear to intertwine with the NeuN and synaptophysin-containing swellings. MAP2 and Aβ-labeling are partly colocalized, and this may be consistent with the model of Takahashi et al. [67, 68], who show that Aβ accumulates at the distal ends of apical dendrites of the CA1 pyramidal neurons. We were not able to label the Aβ with our postembedding immunogold, but Takahashi et al. (2010) [67] used a pre-embedding immunogold method to show that the Aβ accumulates within dendrites in the CA1 region; the associated dendritic structures shown by them would be difficult to identify without the accompanying labeling, so we were not able to identify the specific accumulations in the postsynaptic structures with our ultrastructural studies.

These novel puncta were first observed in 3-month old DPP6-KO mice with increasing accumulation during subsequent development. In contrast, they were first observed in 6-month old WT mice. The pattern of accumulation is similar to that seen for Aβ plaques and tangles in AD in humans, where some are seen in the younger ages, accumulating with aged. The earlier and more substantial appearance of these puncta in the DPP6-KO suggest that DPP6 has a role to prevent puncta formation and deposit and prevents associated neurodegeneration. This also is supported by the presence of greater colocalization of a number of AD marker proteins in the puncta of DPP6-KO mice compared to WT. DPP6 could therefore play a potential role in the early clinical diagnosis and treatment of AD [13].

At the ultrastructural level, the NeuN^+^ swellings resemble various structures described as Lewy bodies or Lewy pathologies in the brains of humans with Parkinson’s disease and dementia [2, 15, 19, 46, 58, 61]; these structures are described as inclusions in neuronal somas and neurites in several brain structures including the substantia nigra, locus coeruleus, nucleus basalis of Meynert, dorsal vagus nucleus, cingulate cortical gyrus, hippocampus, etc. Similar to our described swellings, the structures in the literature often are filled with a variety of vesicular, tubulovesicular, and fibrous structures, including abnormal, deteriorating (dysmorphic) mitochondria and other organelles (described as “potentially damaged, distorted organelles” by Shahmoradian et al., 2019 [58]). Most notably, many of these published electron micrographs show a large accumulation of fibrous/filamentous material interspersed with granular material, again very similar to what we have described here for our NeuN^+^ swellings. Forno (1996) [19] presents an image with 2 large oval neurite swellings somewhat similar to ours, although their filamentous centers are more well-organized.

A number of immunocytochemical studies support the involvement of abnormal, enlarged presynaptic terminals in aging diseases. Lewy structures have been shown to contain α-synuclein [2, 12, 58, 61, 72]. Similarly, we describe a strong, partial colocalization of α-synuclein with our NeuN^+^ large puncta with light microscopy (LM). D’Andrea et al. (2001) [12] show images of circular, “extracellular” “Lewy body-like” structures labeled with MAP2 and α-synuclein in the substantia nigra of humans with Parkinson’s disease; again, we describe a partial colocalization of these 2 proteins associated with our NeuN^+^ large puncta. In humans with dementia with Lewy bodies, Kramer and Schulz-Schaeffer [28] found that the neurodegeneration associated with this dementia may be due mainly to α-synuclein that forms presynaptic aggregates associated with syntaxin and synaptophysin and this phenomenon is independent of the few, large juxtanuclear, neuronal inclusions that are also α-synuclein-positive (only the latter defined here as Lewy bodies). And in a mouse model of dementia with Lewy bodies that bears a mutation of α-synuclein, synaptic/neuritic pathology becomes particularly prevalent in the neuropil of the hippocampus at 4 months of age and increases at 8 months of age [34]. All these studies support our finding of abnormal, enlarged synaptic terminals with associated α-synuclein in the hippocampus in the brain of mice.

The literature also supports our finding that the NeuN^+^ presynaptic structures have strong labeling for synaptophysin and chromogranin A. The structures described as Lewy bodies by Nishimura et al. [46] contain both synaptophysin and chromogranin A, as we describe for our NeuN^+^ puncta/swellings, and they note that “Synaptic vesicles are thus presumably related to the formation of Lewy body.” And Wakabayashi et al. [73] found that Lewy bodies in several regions of the brain and in sympathetic ganglia of humans with Parkinson’s disease were primarily in axons, colocalizing with synaptophysin. Interestingly, labeling for synaptophysin and chromogranin A, along with A4 amyloid, also are found in the senile plaques of humans with AD [8]. Of course, the presence of chromogranin A in amyloid β plaques is well established for human AD as well as for mouse models of AD (reviewed in Willis et al. [76]). Again, this is similar to our findings of synaptophysin, chromogranin A, and Aβ amyloid in our NeuN^+^ large puncta, and this is consistent with our discussion of the relationship of the Aβ and the NeuN large puncta and the Takahashi papers, described above.

We also found an increase in APP in the NeuN + puncta of the DPP6-KO mice. Since APP is a component of the presynaptic membrane [29, 75], perhaps increased APP in DPP6-KO mice contributes to the increase in puncta size. Overexpression of APP results in increases in mammalian spine synapses [31, 74], and overexpression of an APP homolog in neuromuscular junctions in *Drosophila* causes a dramatic increase in the presynaptic membrane, which then expands out as buds from the main membrane [71].

Does the accumulation of a mass tangle of filaments in the NeuN^+^ swellings include modified tau filaments? This is not clear at this point, since we have corroborated with LM and EM only the full colocalization of NeuN and synaptophysin within the central region of the swellings and their mass of filaments. Immunofluorescence shows that there is a partial overlap of pTau labeling with the NeuN^+^ puncta, and which extends also in areas around the NeuN^+^ puncta; unfortunately, we were unable to label for the pTau with EM immunogold. This partial association of the tau as well as Aβ amyloid with the NeuN^+^ puncta supports the findings of Takahashi et al. [67], who found that these 2 pathologies are in the postsynaptic processes in the CA1 area. The structure of tau filaments has been described [18, 39, 70], and ultrastructural studies reveal that these filaments are distributed mainly in neuron somas and dendrites [1, 27, 38, 70], while their presence in the presynaptic compartments is less clear [1, 38, 78]. In studies of mutant mice expressing human tau, several studies [38, 54, 62, 70] found swollen myelinated axon portions called “spheroids” filled with a mixture of neurofilaments, and having labeling for tau. While these latter studies did not find tau accumulations in axon terminals, in general, dystrophic, swollen axonal terminals may be associated with various neuronal pathologies, and perhaps are due to disorders in axonal transport. For dementia, the axonopathy may be due to the effects of abnormal tau protein accumulation, as discussed by Lin et al. (2003) [38] and Terwel et al. [70]. Thus, ultrastructural studies of the brain of patients with Alzheimer’s presenile dementia found dendritic and axonal processes that were swollen with neurofilaments [69]. Swollen, unmyelinated “spheroids” filled with neurofilaments and tubulin have been found in the dentate gyrus of the hippocampus of patients with frontotemporal dementia; these contained only a few tau filaments [78]. Light microscope localization of synaptophysin indicated that they might be presynaptic structures; however, EM studies showed that these structures lacked synaptic vesicles and synapses. Perhaps these are equivalent to the later stage swellings in our study in which synaptic vesicles are no longer present. Based on these previous studies combined with our findings, it is likely that the swellings in the CA1 in our study are filled with neurofilaments, perhaps containing tau filaments as well.

The distribution of NeuN-swellings does not seem to correlate with a specific pattern of innervation. The clusters of large NeuN^+^ puncta are found in the CA1 stratum radiatum, oriens and lacunosum-moleculare, and each large punctum is centered around a swollen presynaptic terminal that is probably excitatory, glutamatergic since there is substantial labeling for VGluT1 on the periphery of the NeuN^+^ punctum, and ultrastructural analysis indicates that the swellings have peripheral, asymmetric synapses on postsynaptic spines. Interestingly, we also have found similar clusters of large, NeuN and synaptophysin-positive puncta in the outer layer of parts of the olfactory cortex, including the piriform cortex and adjacent piriform-amygdala area ventral to this (Additional file 2: 2C,D). Both the hippocampus CA1 and this part of the cortex have a roughly similar basic structural organization, especially centered on the apical dendrites of pyramidal neurons [51, 59]; and adjacent, associated areas of the cortex (entorhinal, perirhinal) along with CA1 are the earliest targets of neurofibrillary tangles associated with AD [42]. The NeuN^+^ clusters in the olfactory cortical areas could originate from axonal input from local pyramidal neurons, e.g., the extensive axon collaterals from pyramidal neurons of the piriform cortex [24], or perhaps from the olfactory bulb afferents, which specifically innervate the outer layer. However, there doesn’t seem to be any one particular, major excitatory innervation of the CA1 that ends in all of its layers. An alternative source of the large NeuN^+^ puncta, other than a glutamatergic input, could be dopaminergic, since the CA1 region has the highest dopaminergic innervation of the hippocampus [16]; but labeling with antibodies to tyrosine hydroxylase and vMAT2 did not show any notable colocalization with the NeuN^+^ puncta (data not shown). Nor was there any particular association of the NeuN^+^ puncta with GAD67; so, a particular inhibitory neuron source is not likely. Perhaps the arrangement of NeuN^+^ clusters is not tied to a particular innervation pattern but reflects more a local pattern of cells in CA1.

**Fig. 8:**
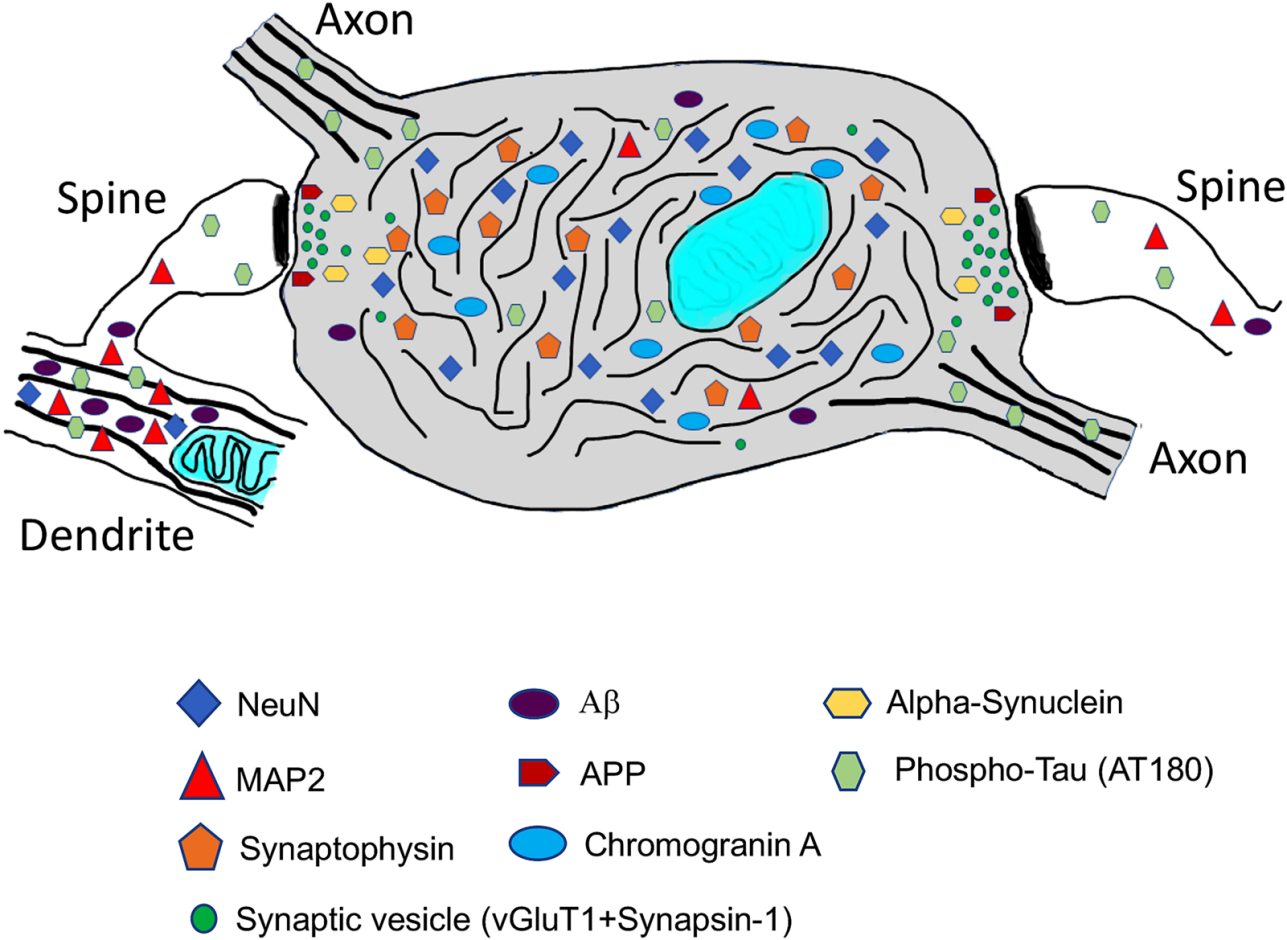
A summary drawing of a NeuN + punctum showing how the various synapse-related and AD-related proteins appear to be arranged. It is based on both the data presented and the published literature. It is portrayed as a presynaptic terminal swelling that forms synapses with two postsynaptic spines (black band represents the postsynaptic density); the left spine is shown extending from its parent dendrite. Microtubules in the axon and dendrite are illustrated with thick black lines and neurofilaments in the swelling are illustrated with thin black lines. The dendrite has a normal mitochondrion (teal color) with distinct cristae (curved lines) while the mitochondrion in the swelling is shown as a dysmorphic (deteriorating) one with indistinct cristae. The main portion of the abnormal terminal swelling is filled with NeuN, synaptophysin, and chromogranin A, while other proteins are concentrated more on the periphery, either in the dwindling presynaptic terminal active zones or the associated postsynaptic structures

A summary diagram of a NeuN + punctum (Fig. 8) shows how the various synapse-related and AD-related proteins appear to be arranged, based on both the data presented here and the published literature discussed above. It shows how the main portion of the abnormal terminal swelling is filled with NeuN, synaptophysin, and chromogranin A, while other proteins are concentrated more on the periphery, either in the dwindling presynaptic terminal active zones or the associated postsynaptic structures.

### DPP6 may play an important neuroprotective role in the prevention of neurodegeneration

Aging is the most important risk factor for the development of neurodegenerative disease and most neurodegenerative disorders manifest in the elderly [26]. A key region of the aging brain associated with neurodegeneration is the hippocampus. In fact, Maruszak and Thuret (2014) [42] note that hippocampal “…dysfunction is believed to underlie the core feature of the disease-memory impairment…” in AD. Our description here of a novel structure in the hippocampus that is observed natively in the aging mouse brain, but which develops sooner and at higher frequency in DPP6-KO mice suggests that DPP6 may normally play an important role in synapse maintenance. Abnormal, enlarged presynaptic terminals in aging diseases are likely associated with neurodegeneration.

## Supplementary information

Additional file 1: Fig. S1 Immunogold labeling of swellings (N) with NeuN (20 nm) in the deep region of the CA1 (deep stratum radiatum and adjacent lacunosum-moleculare) of the hippocampus of a DPP6-KO 12-month old mouse. In **a** and **b**, note the presynaptic active zones (p) that are in the periphery of the swelling and form synapses with postsynaptic spines (sp). Ad, apical dendrites of CA1 pyramidal neurons; m, dysmorphic (deteriorating) mitochondria in the center of the swelling in **C**. Scale bar is 500 nm

Additional file 2: Fig. S2 **A, B**: In the CA1 region of the hippocampus of 12-month old DPP6-KO mice, the immunofluorescence (IF) shows that NeuN (green, 1:500, Millipore Sigma) labeling in the puncta is not colocalized with either GAD67 (**A**. red, 1:2000, Abcam) or ubiquitin (**B**, red, 1:1000, Abcam). Scale bar = 50 μm. **C**: NeuN + puncta found in the piriform cortex. In 12-month old DPP6 mice, IF shows NeuN + puncta located in the piriform cortex. Scale bar = 1 mm; the pirform cortex is magnified in the image on the right. Nuclei were counterstained with DAPI in blue
