# Supplementary figures and images for: A novel structure associated with aging is augmented in the DPP6-KO mouse brain
Source: Acta Neuropathol Commun. 2020 Nov 23;8:197. doi: 10.1186/s40478-020-01065-7 (PMC7682109; doi:10.1186/s40478-020-01065-7)

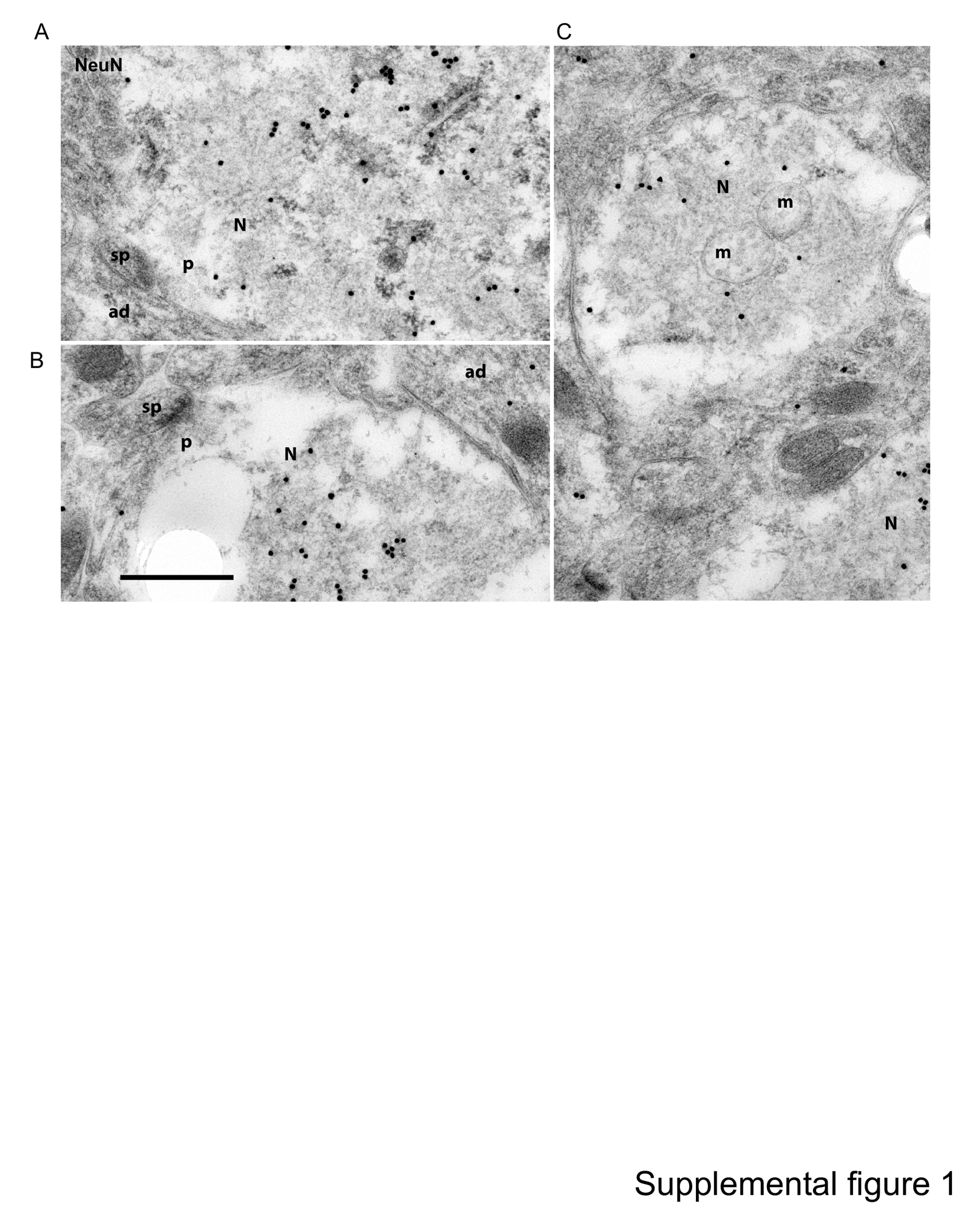

Supplement: Supplementary file 1 — Immunogold labeling of swellings (N) with NeuN (20 nm) in the deep region of the CA1 (deep stratum radiatum and adjacent lacunosum-moleculare) of the hippocampus of a DPP6-KO 12-month old mouse. In a and b, note the presynaptic active zones (p) that are in the periphery of the swelling and form synapses with postsynaptic spines (sp). Ad, apical dendrites of CA1 pyramidal neurons; m, dysmorphic (deteriorating) mitochondria in the center of the swelling in C. Scale bar is 500 nm [file 40478_2020_1065_MOESM1_ESM.tif]

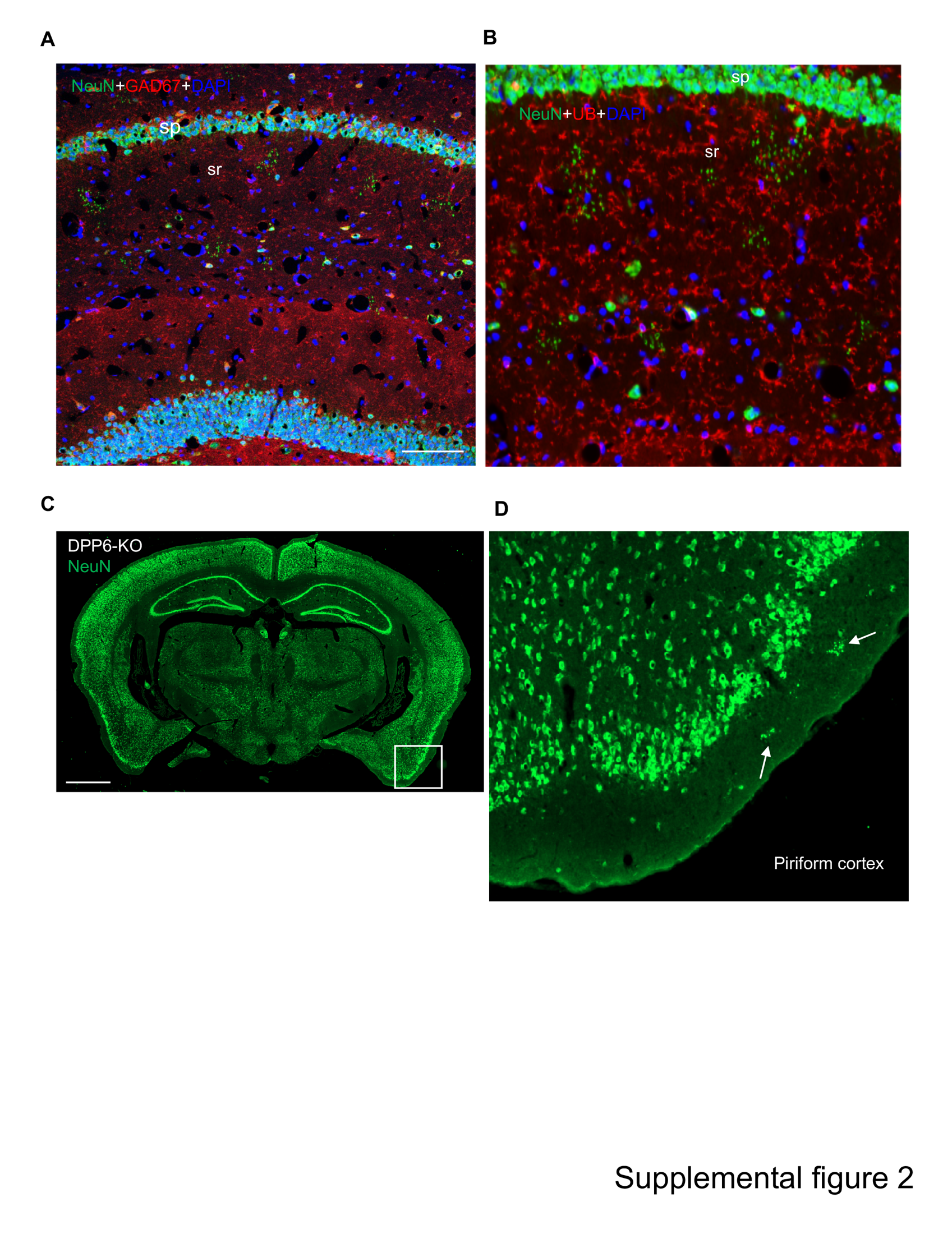

Supplement: Supplementary file 2 — A, B: In the CA1 region of the hippocampus of 12-month old DPP6-KO mice, the immunofluorescence (IF) shows that NeuN (green, 1:500, Millipore Sigma) labeling in the puncta is not colocalized with either GAD67 (A. red, 1:2000, Abcam) or ubiquitin (B, red, 1:1000, Abcam). Scale bar = 50 μm. C: NeuN + puncta found in the piriform cortex. In 12-month old DPP6 mice, IF shows NeuN + puncta located in the piriform cortex. Scale bar = 1 mm; the pirform cortex is magnified in the image on the right. Nuclei were counterstained with DAPI in blue [file 40478_2020_1065_MOESM2_ESM.tif]
